# Validation of transcriptome signature reversion for drug repurposing in oncology

**DOI:** 10.1093/bib/bbac490

**Published:** 2022-11-29

**Authors:** Karel K M Koudijs, Stefan Böhringer, Henk-Jan Guchelaar

**Affiliations:** Department of Clinical Pharmacy and Toxicology, Leiden University Medical Center (LUMC); 2333 ZA Leiden, The Netherlands; Department of Clinical Pharmacy and Toxicology, Leiden University Medical Center (LUMC); 2333 ZA Leiden, The Netherlands; Department of Biomedical Data Sciences, Leiden University Medical Center (LUMC); 2333 ZA Leiden, The Netherlands; Department of Clinical Pharmacy and Toxicology, Leiden University Medical Center (LUMC); 2333 ZA Leiden, The Netherlands

**Keywords:** Drug repositioning, Systematic validation, Gene expression, Bioinformatics, Oncology

## Abstract

Transcriptome signature reversion (TSR) has been extensively proposed and used to discover new indications for existing drugs (i.e. drug repositioning, drug repurposing) for various cancer types. TSR relies on the assumption that a drug that can revert gene expression changes induced by a disease back to original, i.e. healthy, levels is likely to be therapeutically active in treating the disease. Here, we aimed to validate the concept of TSR using the PRISM repurposing data set, which is—as of writing—the largest pharmacogenomic data set. The predictive utility of the TSR approach as it has currently been used appears to be much lower than previously reported and is completely nullified after the drug gene expression signatures are adjusted for the general anti-proliferative downstream effects of drug-induced decreased cell viability. Therefore, TSR mainly relies on generic anti-proliferative drug effects rather than on targeting cancer pathways specifically upregulated in tumor types.

## Introduction

Transcriptome signature reversion (TSR) has been extensively used with the aim to discover new indications for existing drugs (i.e. drug repurposing, drug repositioning) for various cancer types [1]. TSR relies on the assumption that a drug that can revert gene expression changes induced by a disease back to its original, i.e. healthy, state is likely to be therapeutically active in treating the disease.

The typical TSR drug repositioning study proceeds according to the following approach: first, the genes upregulated and downregulated in the tumor tissue as compared with the adjacent ‘healthy normal’ tissue (tumor signature) are identified using differential gene expression analysis. Next, either the Connectivity Map database or its successor the Library of Integrated Network-Based Cellular Signatures (LINCS) L1000 database is screened for gene expression signatures of drugs (drug signatures), which reverse the differentially expressed genes (DEG) identified in the first step in the opposite direction [2, 3]. Finally, the top drug candidates are tested on one or more cancer cell lines and/or in an animal model of the same tumor type as the tumor signature. Using this procedure, several studies have elicited drugs with anticancer activity against specific tumor types [4–11], suggesting that TSR has a high predictive ability for prioritizing drug repurposing candidates. The best available systematic evidence for the concept of TSR in oncology was published by Chen *et al.* [6], which demonstrated that the potential to revert the gene expression signatures of breast, liver and colorectal tumors is associated with the median half maximal inhibitory concentration (IC_50_) of compounds tested in a single cell line of that tumor type.

However, drugs identified using TSR may be targeting the downstream proliferative ‘effect’ rather than the upstream ‘cause’ of the proliferative phenotype characterizing a specific cancer type. Indeed, it has been shown that drugs that decrease cell viability show similarity in gene expression perturbation signatures, which is linked to transcription factors regulating cell death, proliferation and division time [12]. In other words, a stronger expected inversion of a tumor gene expression signature based on the drug signature could perhaps only be a proxy for the general anti-proliferation effect of a drug and not specifically targeting the tumor (sub)type under investigation. This would imply that TSR as currently implemented is less useful for drug repurposing than claimed, as it would not provide any specificity in prioritizing which drugs may be effective against a particular tumor type. Alternatively, it seems appropriate to remove the downstream gene expression effects related to decreased cell viability from the drug signatures in order to increase the predictive utility of TSR.

In this study, we aim to comprehensively validate the concept of TSR using a series of 18 different solid tumor types with at least four cell lines per tumor type and 400+ drugs for each of these tumor types based on the recent release of the PRISM repurposing data set [13]. In addition, we repeated the analysis using drug signatures from which downstream effects of reduced cell viability were removed to determine if this step increases or decreases the predictive power of TSR for selecting whether a drug has the potential to be repurposed against a particular tumor type.

## Materials and methods

### Study design

**Figure 1:**
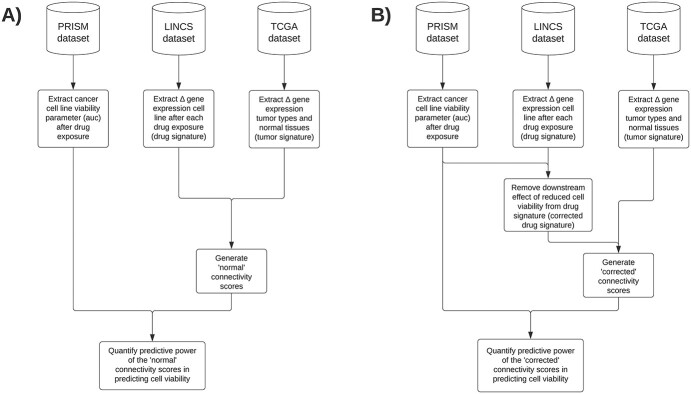
Graphical illustration of the main method of generating the default connectivity scores (**A**) and corrected connectivity scores (**B**) and using it to quantify the predictive power in prediction cell viability.

To answer the main research objective of validating the predictive utility of connectivity scores for prioritizing drug repurposing candidates, we combined publicly available data from the PRISM, LINCS and The Cancer Genome Atlas (TCGA) databases (Figure 1). Data acquisition, data inclusion/exclusion criteria and summarization steps are described below in more detail.

### Cancer cell line viability data

As of writing, the PRISM repurposing data set constitutes the largest data available on cancer cell line viability, significantly more than earlier publicly released high-throughput databases (i.e. GDSC1000 and CTRPv2) [13] . The data in PRISM were generated in two stages: in the first screening stage, 578 cancer cell lines were screened with up to 4518 drugs at a single concentration of 2.5 μM, and in the second screening stage, the 1448 of the most active drugs at this single concentration were retested at eight concentrations ranging from 610 pM to 10 μM. As the only differences in effectivity between cell lines can be expected for these active drugs, we focus the analysis on data from the second stage. These data were downloaded on 14 June 2021 from the PRISM Repurposing 21Q2 release available on ‘depmap.org’ (release with filenames ‘secondary-screen-dose-response-curve-parameters.csv’ and ‘secondary-screen-cell-line-info.csv’). Four parameters that result from fitting the dose response curve were available, called ‘slope’, ‘auc’, ‘ec50’ and ‘ic50’, in the data set. The most natural choice would be to use the half maximal inhibitory concentration (IC_50_) as this was used in the paper of Chen B *et al.*; however, the IC_50_ was only estimable in 51% of experiments because of the limited concentration range used in the experiments. The half maximal effective concentration (EC_50_) was also not a great choice to compare drugs with each other because of the expected differences in the maximal response between drugs and its high variability (ranged from 1 × 10^−7^ to 4 × 10^304^). The slope has a wide data range, which includes negative numbers (−4891 to +18 537) and is therefore difficult to interpret. The dose–response area under the curve (AUC) therefore appeared to be the best option as it has the narrowest range (0.004174 to 4.889162) of all parameters with a relatively simple interpretation: it represents the fraction of cells left after drug exposure averaged over all the tested concentrations normalized to cells receiving no drug treatment, such that AUC values below 1 indicate sensitivity to treatment. The nonnegative nature of the distribution also makes it possible to log-transform, making it easier to include in statistical models and to display in figures.

### Drug gene expression perturbation data

Drug signatures, based on data generated by the LINCS Program [3], were calculated with the goal of estimating the effect in the average tumor cell line at 10 μM for 6 h and 24h. This concentration was chosen because it is the most common concentration used in all experiments and the concentration is at the upper limit of the concentrations tested in the second phase of PRISM. Experiments that tested concentrations other than 10 μM (37% of total) were removed. Additionally, drugs that were tested in less than 5 distinct cell lines were removed because of small sample size, implying poor generalizability. Experiments with drugs incubated for 6 hours and 24 hours were analyzed separately. For each data subset (i.e. 6- and 24-h experiments), a separate linear mixed model was fitted on each of the 978 landmark genes with treatment as a categorical variable using DMSO control vehicle as the reference level and cell line and plate as two separate random effects, allowing for correlation. To create a consensus drug signature between the 6- and 24-h drug signatures, the fold change (FC) and standard error estimates of 6 h and 24 h were combined using meta-analysis using the ‘rma’ function in the ‘metafor’ package (version 3.0-2).

### Cancer tissue gene expression signature

TCGA RNA-seq HTSeq counts data and associated meta-data were downloaded from gdc.xenahubs.net (version 12 November 2017; 11 538 samples in total) [14]. Twenty-two TCGA projects had enough tumor and adjacent normal samples to run a differential expression analysis contrasting the two tissue types (Supplementary Table S1). Genes with a mean log_2_-FC (Counts + 1) expression below 6 were removed, so that only the 13 386 most highly expressed genes remained (Supplementary Figure S1).

To create the tumor gene expression signatures, the samples of each tumor type were first normalized using the upperquartile method using the ‘calcNormFactors’ function from ‘edgeR’ package (version 3.35.1). Next the data were analyzed using the ‘voom’ function from the ‘limma’ package (version 3.49.4), with a fixed effect for the tumor versus normal contrast. To create the ‘meta-analysis mean tumor signature’ used in Figure 3D, the log_2_-FC estimates and standard errors of each tumor type, the rma function in the metafor package (version 3.0-2) was used with all default options.

### Estimating the mean normalized AUC (mnAUC)

Because each drug in PRISM is not always tested in exactly the same set of cell lines, a linear mixed model was used to separate the effect of cell lines and drugs. This step summarizes the AUC into an mnAUC. The interpretation of the mnAUC is the same as the original AUC, but it now represents the average fraction of cells left after drug exposure of a set of cell lines. First a linear mixed model was fitted using the ‘lme4’ package (version 1.1-27.1) using the following formula:

$$ \log \left(\mathrm{auc}\right)\sim 1+\left(1|\ \mathrm{drug}\_\mathrm{name}\right)+\left(1|\mathrm{cellLine}\_\mathrm{id}\right). $$

Then, the mnAUC was calculated using the following formula:

$$ {\mathrm{mnAUC}}_{\mathrm{drug}=\mathrm{i}}={\mathrm{e}}^{\mathrm{Intercept}+\mathrm{drug}\_{\mathrm{ranef}}_{\mathrm{i}}}, $$

where drug_ranef_ is the posterior Bayes estimates for the respective drug in the mixed model. This calculation was done for all drugs and cell lines at the same time and for the subsets of experiments that used cell lines that are associated with a particular TCGA tumor type.

Supplementary Table S1 lists the selection and number of PRISM cancer cell lines mapped to each tumor type.

### Removing downstream impact of reduced cell viability from drug signatures

It has been observed that drugs that strongly decrease cell viability share a common gene signature response linked to transcription factors regulating cell death, proliferation and division time [12]. The goal of this procedure is to remove the downstream effects of the drug action (i.e. cell death, reduced proliferation and division time) from the drug’s gene expression signature. If a gene is consistently upregulated or downregulated in the presence of drugs with different mechanisms of action, which strongly decreases cell viability, this gene is unlikely to be linked to the unique mechanism of action of a specific drug. The second-order effect of the strongly decreased cell viability is approximated by regressing the log_2_-FC after drug exposure of each gene after (*y*-axis) on the logarithm of the mnAUC (*x*-axis). The corrected DE is taken to be the residual, i.e. the part of the variation in differential expression not explained by the mnAUC (Supplementary Figure S2). This process was repeated for all 978 genes measured by the LINCS array, separately for the 6- and 24-h drug exposure subsets of the data. To get the corrected combined 6–24 h drug signatures, the corrected 6- and 24-h drug signatures were combined using meta-analysis as described previously.

### Calculating connectivity scores

To calculate a connectivity score, one first needs to create a tumor signature. The first step is to remove any genes that were not measured in the LINCS microarray data, leaving 952 overlapping genes. The next step would be to only include genes that pass a certain threshold. The paper of Chen *et al*. [6] only included genes that were differentially expressed above 1.5 log_2_-FC or below -1.5 log_2_-FC in combination with adj. P-value below 0.001, resulting in between 65 and 83 genes in the 3 tumor signatures. Using the same criteria for the 18 tumor signatures included in this research would result in between 3 and 176 genes (Supplementary Table S1). To minimize differences in the size of the tumor signature between tumor types and explore the effect of variation in tumor signature selection, we decided to test four different scenarios: use the 50, 100 and 150 most statistically significant genes DEG (scenarios 1–3) and use the same method from Chen *et al*. to make a direct comparison easier (scenario 4).

The connectivity scores were calculated using both the original method using the ‘connectivityScore’ function in the ‘PharmacoGx’ package (version 2.5.2) and the newer version ‘cmap_score_new’ available from https://github.com/Bin-Chen-Lab/RGES. The benefit of the newer version is that it does not produce any connectivity scores of 0 in specific situations as described in the paper of Chen et al. [6], which could bias the results in correlation analyses. After validating that this newer version of connectivity scoring worked similarly to the original connectivity score without producing any connectivity scores of 0 (Supplementary Figure S3), we decided to use the newer version for our main results.

### Gene set enrichment analysis (GSEA)

GSEA was performed using Enrichr, an enrichment analysis web-based tool providing various types of visualization summaries of collective functions of gene lists [15]. Using this website, the gene sets of interest were uploaded to the gene symbol form field available at https://maayanlab.cloud/Enrichr/ in April 2022.

### Statistical analyses

All correlations mentioned in the results used the nonparametric Spearman correlation (R function ‘cor.test’, ‘method’ = ‘spearman’). This nonparametric method is used to obviate the assumption that the relationship between the variables is linear and has the advantage that it is invariant to monotone transformations of the variables (i.e. taking the logarithm of a variable does not change the correlation coefficient).

## Results

### Data retrieval and preprocessing

**Figure 2:**
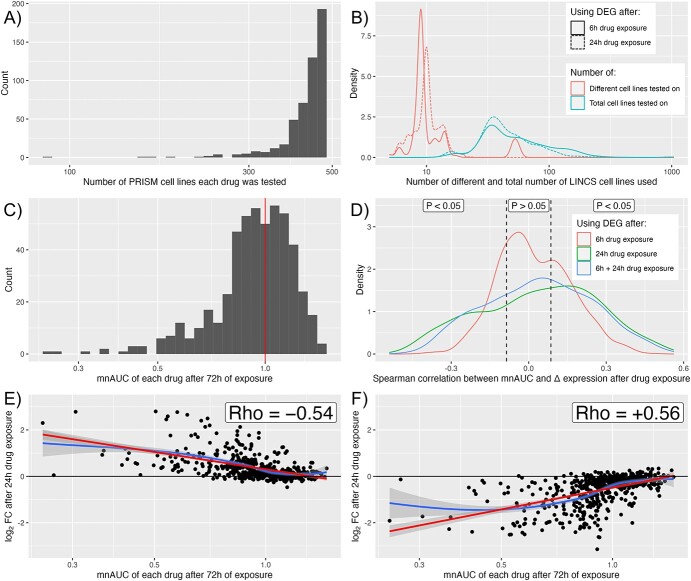
Data on number of cell lines each of the 506 included drugs were tested on in PRISM and LINCS, the distribution of the mnAUC and the association between mnAUC and differential expression after drug exposure. (**A**) Histogram of the number of cell lines each drug was tested on in PRISM. (**B**) Number of different cell lines, and total number of cell lines, each drug was tested on in LINCS. The data are separately presented for both 6 h exposure and 24 h exposure subsets of the data. (**C**) Distribution of the mnAUC. The solid red line represents the ‘null’ value, i.e. no effect compared with control experiments for the average cell line. (**D**) Distribution of the Spearman rho correlation coefficient for the 6 h, 24 h and the combined 6 + 24-h drug exposure gene expression signatures. The dashed black lines at −0.086 and + 0.086 represent the critical rho values, below and above which the association is statistically significant. (**E**, **F**) Example of a gene [GADD45A, (E)] whose expression after 24-h drug exposure is strongly negatively correlated with mnAUC and of a gene [POLE2, (F)] whose expression after 24-h drug exposure is strongly positively correlated with mnAUC. The blue line is the LOESS moving average and the red line is the linear model fit. *Note:* the *X*-axes of (A–F) are logarithmic.

PRISM and LINCS share 506 drugs that fit the inclusion criteria of the differential expression of drugs after drug exposure being measured in at least five cell lines. Of these included drugs, the cell viability was tested on between 89 and 479 cancer cell lines with a median of 445 (Figure 2A), and the differential expression after exposure to the drug was tested on a median of 10 different cell lines with on average 4.2 replications per cell line, resulting in a median total sample size of 42 (Figure 2B).

The mnAUC was centered around 1 (i.e. no effect versus control in the average cancer cell line) for the majority of drugs, with a sizeable minority having an mnAUC below 1, which signifies anticancer efficacy (Figure 2C). The association of the mnAUC with the DEG after 6 h, 24 h and 6 + 24 h shows that the majority of genes are statistically significantly associated with mnAUC at a significance level of *P* < 0.05 (Figure 2D), with most (75%) of genes being affected after 24-h drug exposure and the fewest genes (56%) after 6h of drug exposure. The combined 6 + 24-h drug signatures scored in between at 70% of genes affected.

Figure 2E illustrates an example of a strong negative correlation and Figure 2F illustrates an example of a strong positive correlation after 24-h drug treatment. In both cases, the DEG seem normally distributed to good approximation (i.e. centered around log_2_-FC = 0) from mnAUC >1, but starts to increase and decrease, respectively, when the mnAUC decreases below 1. Note that a positive correlation in this context implies that the gene is downregulated in the presence of drugs that decrease cell viability, whereas a negative correlation implies that the gene is upregulated. Drugs with an mnAUC >1 have on average far fewer DEG than those with mnAUC <1 (Supplementary Figure S4), which reinforces why the drugs with mnAUC >1 could be considered the control group.

### Inverse association between genes upregulated in cancer and downregulated after drug-induced reduced cell viability

**Figure 3:**
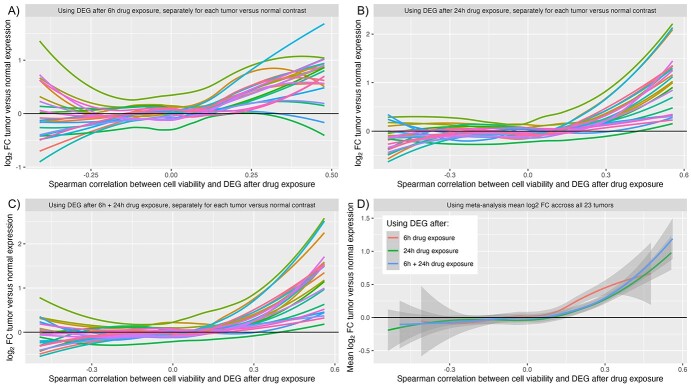
The log_2_-FC of each tumor tissue versus adjacent normal tissue contrast plotted against the Spearman rho of the correlation between mnAUC and DEG after drug exposure. Panels (**A**–**C**) do this, respectively, for the 6 h, 24 h and the combined 6 + 24-h drug perturbation signatures separately for each of the 23 tumor types included in the analysis. Panel (**D**) instead uses the meta-analysis mean of the log_2_-FC contrast, with each color representing a different drug perturbation signature subset.

When the log_2_-FC of the tumor tissues versus the adjacent normal tissues is plotted against the correlation coefficient between the mnAUC and the DEG after 6, 24 and 6 + 24 h, it can be seen that genes that are downregulated after exposure to drugs that decrease cell viability (from around rho > 0.15) are on average upregulated in most tumor tissues (Figure 3). Supplementary Table S2 lists correlation estimates and *P*-values: all correlation coefficients are positive and at least for 19 out of 23 tumor types the correlation is statistically significant for all 3 types of drug signatures used (i.e. 6 h, 24 h and 6 + 24 h combined).

To find out if there is a common link between the set of genes that are both frequently upregulated in cancer and strongly positively associated with mnAUC after drug exposure (thus downregulated in the presence of drugs that decrease cell viability, see Figure 2F), we performed gene set enrichment analysis using the 25 genes with the most positive correlations with mnAUC (rho > 0.42, Supplementary Table S3). Results from the KEGG 2021 pathway database indicate the genes have the strongest association with the ‘Cell cycle’ pathway (adj. *P*-value: 3 × 10^−7^), with other strong contenders being ‘DNA replication’ (adj. *P*-value: 3 × 10^−4^), ‘Mismatch repair’ (adj. *P*-value: 6 × 10^−3^), ‘Base excision repair’ (adj. *P*-value: 9 × 10^−3^) and ‘Cellular senescence’ (adj. *P*-value: 9 × 10^−3^).

### Predictive power of normal and corrected connectivity scores

For the 18 TCGA tumor types PRISM cell lines could be matched to, we tested whether the connectivity score calculated using the tumor’s gene expression signatures was predictive of the anticancer effect to cell lines associated with that tumor type (Tables 1 and 2).

**Table 1:** Spearman correlation coefficients between the mnAUC of a drug in cell lines of the corresponding tumor and the connectivity scores calculated using various methods of selecting the most statistically significant DEG in the corresponding tumor tissue using the drug signatures not corrected for the overall association between the DEG after drug exposure and mnAUC

TCGA project	Using 50 most statistically significant genes		Using 100 most statistically significant genes		Using 150 most statistically significant genes		Using genes >1.5 log_2_FC and adj. *P* < 0.001	
BLCA	+0.22 (*P* = 8 × 10^−7^)	^*^^*^^*^	+0.24 (*P* = 8 × 10^−8^)	^*^^*^^*^	+0.28 (*P* = 2 × 10^−10^)	^*^^*^^*^	+0.24 (*P* = 5 × 10^−8^)	^*^^*^^*^
BRCA	+0.10 (*P* = 0.02)	^*^	+0.12 (*P* = 0.007)	^*^^*^	+0.13 (*P* = 0.005)	^*^^*^	+0.16 (*P* = 0.0003)	^*^^*^^*^
CHOL	−0.02 (*P* = 0.69)		−0.03 (*P* = 0.44)		+0.02 (*P* = 0.61)		+0.03 (*P* = 0.45)	
COAD	+0.01 (*P* = 0.77)		+0.14 (*P* = 0.002)	^*^^*^	+0.18 (*P* = 3 × 10^−5^)	^*^^*^^*^	+0.01 (*P* = 0.88)	
ESCA	+0.09 (*P* = 0.03)	^*^	+0.13 (*P* = 0.003)	^*^^*^	+0.13 (*P* = 0.003)	^*^^*^	+0.17 (*P* = 0.0001)	^*^^*^^*^
GBM	+0.22 (*P* = 3 × 10^−7^)	^*^^*^^*^	+0.17 (*P* = 0.0002)	^*^^*^^*^	+0.27 (*P* = 4 × 10^−10^)	^*^^*^^*^	+0.24 (*P* = 6 × 10^−8^)	^*^^*^^*^
KICH	−0.18 (*P* = 5 × 10^−5^)	^*^^*^^*^	−0.11 (*P* = 0.01)	^*^	−0.05 (*P* = 0.31)		−0.01 (*P* = 0.88)	
KIRC	0 (*P* = 0.93)		0 (*P* = 0.91)		+0.01 (*P* = 0.84)		+0.10 (*P* = 0.02)	^*^
KIRP	+0.12 (*P* = 0.007)	^*^^*^	+0.07 (*P* = 0.14)		+0.12 (*P* = 0.008)	^*^^*^	+0.15 (*P* = 0.0007)	^*^^*^^*^
LIHC	+0.14 (*P* = 0.001)	^*^^*^	+0.14 (*P* = 0.001)	^*^^*^	+0.20 (*P* = 7 × 10^−6^)	^*^^*^^*^	+0.26 (*P* = 2 × 10^−9^)	^*^^*^^*^
LUAD	+0.07 (*P* = 0.10)		+0.23 (*P* = 2 × 10^−7^)	^*^^*^^*^	+0.19 (*P* = 1 × 10^−5^)	^*^^*^^*^	+0.24 (*P* = 5 × 10^−8^)	^*^^*^^*^
LUSC	+0.08 (*P* = 0.06)		+0.13 (*P* = 0.003)	^*^^*^	+0.22 (*P* = 7 × 10^−7^)	^*^^*^^*^	+0.24 (*P* = 4 × 10^−8^)	^*^^*^^*^
PAAD	+0.06 (*P* = 0.18)		+0.09 (*P* = 0.05)		+0.15 (*P* = 0.0005)	^*^^*^^*^	–	
PRAD	+0.13 (*P* = 0.003)	^*^^*^	+0.12 (*P* = 0.007)	^*^^*^	+0.13 (*P* = 0.005)	^*^^*^	+0.17 (*P* = 0.0001)	^*^^*^^*^
SARC	+0.06 (*P* = 0.16)		+0.08 (*P* = 0.06)		+0.11 (*P* = 0.02)		−0.04 (*P* = 0.39)	
STAD	+0.24 (*P* = 8 × 10^−8^)	^*^^*^^*^	+0.21 (*P* = 1 × 10^−6^)	^*^^*^^*^	+0.25 (*P* = 1 × 10^−8^)	^*^^*^^*^	+0.15 (*P* = 0.0006)	^*^^*^^*^
THCA	−0.08 (*P* = 0.08)		−0.05 (*P* = 0.28)		−0.02 (*P* = 0.67)		+0.13 (*P* = 0.003)	^*^^*^
UCEC	+0.14 (*P* = 0.001)	^*^^*^	+0.16 (*P* = 0.0003)	^*^^*^^*^	+0.15 (*P* = 0.0006)	^*^^*^^*^	+0.18 (*P* = 3 × 10^−5^)	^*^^*^^*^

*Note*: ^*^P < 0.05; ^*^^*^P < 0.01; ^*^^*^^*^P < 0.001.

**Table 2:** Spearman correlation coefficients between the mnAUC of a drug in cell lines of the corresponding tumor and the connectivity scores calculated using the 50, 100 and 150 most statistically significant DEG in the corresponding tumor tissue using the drug signatures corrected for the overall association between the DEG after drug exposure and mnAUC

TCGA project	Using 50 most statistically significant genes		Using 100 most statistically significant genes		Using 150 most statistically significant genes		Using genes >1.5 log_2_FC and adj. *P* < 0.001	
BLCA	−0.01 (*P* = 0.86)		+0.04 (*P* = 0.42)		+0.05 (*P* = 0.27)		−0.01 (*P* = 0.81)	
BRCA	+0.02 (*P* = 0.61)		+0.03 (*P* = 0.52)		+0.04 (*P* = 0.42)		+0.04 (*P* = 0.38)	
CHOL	+0 (*P* = 0.94)		−0.12 (*P* = 0.007)	^*^^*^	−0.05 (*P* = 0.25)		+0.03 (*P* = 0.48)	
COAD	+0.02 (*P* = 0.69)		+0.01 (*P* = 0.90)		+0.01 (*P* = 0.74)		−0.02 (*P* = 0.61)	
ESCA	+0.11 (*P* = 0.01)	^*^	+0.11 (*P* = 0.01)	^*^	+0.14 (*P* = 0.001)	^*^^*^	+0.05 (*P* = 0.28)	
GBM	−0.03 (*P* = 0.57)		0 (*P* = 0.95)		+0.05 (*P* = 0.25)		+0.02 (*P* = 0.69)	
KICH	−0.11 (*P* = 0.01)	^*^	−0.15 (*P* = 0.0005)	^*^^*^^*^	−0.10 (*P* = 0.02)	^*^	−0.05 (*P* = 0.22)	
KIRC	−0.03 (*P* = 0.50)		+0.05 (*P* = 0.26)		+0.08 (*P* = 0.08)		+0.05 (*P* = 0.22)	
KIRP	−0.07 (*P* = 0.14)		−0.03 (*P* = 0.49)		−0.02 (*P* = 0.72)		+0.07 (*P* = 0.12)	
LIHC	−0.01 (*P* = 0.85)		−0.03 (*P* = 0.49)		+0.01 (*P* = 0.75)		+0.04 (*P* = 0.39)	
LUAD	−0.05 (*P* = 0.24)		+0.02 (*P* = 0.68)		+0.03 (*P* = 0.55)		−0.01 (*P* = 0.86)	
LUSC	+0.01 (*P* = 0.74)		+0.03 (*P* = 0.48)		+0.04 (*P* = 0.40)		0 (*P* = 0.93)	
PAAD	−0.13 (*P* = 0.003)	^*^^*^	−0.06 (*P* = 0.18)		−0.06 (*P* = 0.17)		–	
PRAD	−0.05 (*P* = 0.31)		−0.03 (*P* = 0.57)		−0.02 (*P* = 0.68)		+0.01 (*P* = 0.86)	
SARC	−0.18 (*P* = 4 × 10^−5^)	^*^^*^^*^	−0.06 (*P* = 0.19)		−0.03 (*P* = 0.46)		−0.08 (*P* = 0.06)	
STAD	+0.10 (*P* = 0.02)	^*^	+0.06 (*P* = 0.15)		+0.06 (*P* = 0.19)		+0.03 (*P* = 0.56)	
THCA	+0.07 (*P* = 0.11)		+0.02 (*P* = 0.65)		−0.08 (*P* = 0.08)		−0.03 (*P* = 0.44)	
UCEC	+0.05 (*P* = 0.31)		+0.03 (*P* = 0.47)		+0.06 (*P* = 0.16)		−0.02 (*P* = 0.73)	

*Note*: ^*^P < 0.05; ^*^^*^P < 0.01; ^*^^*^^*^P < 0.001.

**Figure 4:**
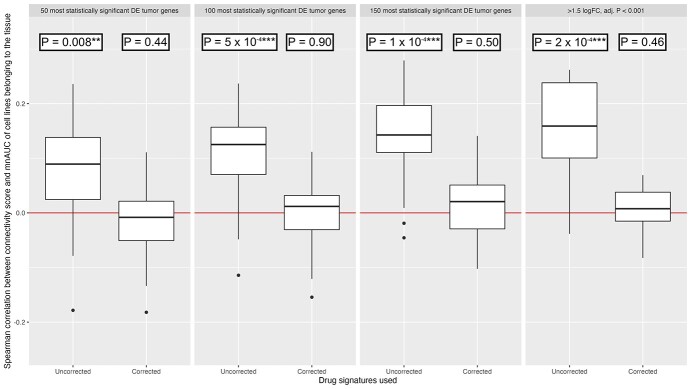
Distributions of the correlation coefficient between the mnAUC of a drug in the cell lines of the corresponding tumor and the connectivity scores calculated using the 50, 100, 150 most statistically significant DEG and using genes above or below 1.5 log_2_FC & adj. *P* < 0.001, in the corresponding tumor tissue. *P*-values presented were generated using the nonparametric Wilcoxon signed rank exact test, testing with the null hypothesis that the median is equal to 0. The red line signifies a Spearman rho of 0, i.e. no association between the connectivity score and mean AUC of the tumor cell lines. Two and three stars signify *P*-values below 0.01 and 0.001, respectively.

Without correcting for the downstream gene expression effects of drug-induced decreased cell viability, there is indeed a statistically significant trend between the connectivity score and the mnAUC of the drug calculated in cell lines belonging to the same tumor type for most tumor tissues, but this trend disappears when the downstream effects of drug-induced decreased cell viability on the drug gene expression profile are removed (Figure 4) by conditioning on the effects seen in other cell lines at the same mnAUC.

Interestingly, without correction the overall association is already highly statistically significant for most tumors using the 50 most statistically significant DEG as input for the connectivity scoring method. This relationship becomes even stronger when 100 or 150 genes are used. Using the method of selecting tumor genes used by Chen *et al*., i.e. only including genes in the tumor signature that have a log_2_-FC above 1.5 or below −1.5 and an adjusted *P*-value below 0.001, produced qualitatively identical results.

Our results so far indicate that the connectivity scores calculated using the ‘uncorrected’ drug signatures do have some power to predict the sensitivity of cell lines of a particular tumor type to a specific drug, as asserted by earlier publications. However, by far the strongest predictor of the mnAUC of a drug in cell lines belonging to a specific tumor type is the mnAUC calculated using ‘all other cell lines’: this produces a median *R*^2^ of 95.5% (Table 3, model 2 column) compared with a median *R*^2^ of 1.6% for the ‘uncorrected’ connectivity score (Table 3, model 1 column). Furthermore, adding the connectivity score as a second predictor to the mnAUC calculated in other cell lines increases the *R*^2^ by a median of 0.01% (Table 3, model 3 column), suggesting almost all the predictive power of the connectivity score is already captured by the overall drug-induced decreased cell viability of the drug in the other cell lines.

**Table 3:** Percentage of variation explained in the mean AUC of cell lines belonging to a specific tumor type by three different linear models. If the correlation coefficient of the connectivity score was negative (i.e. opposite the expected direction), the *P*-values were replaced with 1

TCGA project	Model 1: connectivity score as covariate (R^2^ and *P*-value versus null model)		Model 2: mean AUC calculated using other cell lines as covariate (*R*^2^ and *P*-value versus null model)		Model 3: connectivity score added to model 2 (Increase in *R*^2^ and *P*-value versus model 2)	
BLCA	5.87% (*P* = 3 × 10^−8^)	^*^^*^^*^	97.6% (*P* < 1 × 10^−240^)	^*^^*^^*^	0.004% (*P* = 0.34)	
BRCA	1.74% (*P* = 0.003)	^*^^*^	96.3% (*P* < 1 × 10^−240^)	^*^^*^^*^	0.00005% (*P* = 0.94)	
CHOL	0.0005% (*P* = 1)		91.7% (*P* < 1 × 10^−240^)	^*^^*^^*^	0.07% (*P* = 0.04)	
COAD	2.31% (*P* = 0.0006)	^*^^*^^*^	95.5% (*P* < 1 × 10^−240^)	^*^^*^^*^	0.02% (*P* = 0.17)	
ESCA	0.94% (*P* = 0.03)	^*^	96.8% (*P* < 1 × 10^−240^)	^*^^*^^*^	0.05% (*P* = 0.005)	^*^^*^
GBM	5.78% (*P* = 4 × 10^−8^)	^*^^*^^*^	96.9% (*P* < 1 × 10^−240^)	^*^^*^^*^	0.0004% (*P* = 1)	
KICH	0.10% (*P* = 1)		88.8% (*P* < 1 × 10^−240^)	^*^^*^^*^	0.03% (*P* = 0.22)	
KIRC	0.07% (*P* = 1)		88.8% (*P* < 1 × 10^−240^)	^*^^*^^*^	0.02% (*P* = 1)	
KIRP	0.84% (*P* = 0.04)	^*^	88.8% (*P* < 1 × 10^−240^)	^*^^*^^*^	0.0001% (*P* = 0.94)	
LIHC	3.32% (*P* = 4 × 10^−5^)	^*^^*^^*^	95.2% (*P* < 1 × 10^−240^)	^*^^*^^*^	0.01% (*P* = 0.3)	
LUAD	2.81% (*P* = 0.0001)	^*^^*^^*^	98.7% (*P* < 1 × 10^−240^)	^*^^*^^*^	0.005% (*P* = 1)	
LUSC	3.66% (*P* = 1 × 10^−5^)	^*^^*^^*^	97.1% (*P* < 1 × 10^−240^)	^*^^*^^*^	0.001% (*P* = 1)	
PAAD	1.37% (*P* = 0.008)	^*^^*^	97.3% (*P* < 1 × 10^−240^)	^*^^*^^*^	0.003% (*P* = 0.46)	
PRAD	1.89% (*P* = 0.002)	^*^^*^	89.5% (*P* < 1 × 10^−240^)	^*^^*^^*^	0.03% (*P* = 1)	
SARC	0.59% (*P* = 0.09)		90.1% (*P* < 1 × 10^−240^)	^*^^*^^*^	0.12% (*P* = 1)	
STAD	3.95% (*P* = 7 × 10^−6^)	^*^^*^^*^	95.3% (*P* < 1 × 10^−240^)	^*^^*^^*^	0.06% (*P* = 0.01)	^*^
THCA	0.01% (*P* = 0.84)		95.5% (*P* < 1 × 10^−240^)	^*^^*^^*^	0.01% (*P* = 1)	
UCEC	1.46% (*P* = 0.006)	^*^^*^	96.4% (*P* < 1 × 10^−240^)	^*^^*^^*^	0.04% (*P* = 1)	
Median:	1.6%		95.5%		0.014%	

*Note*: ^*^P < 0.05; ^*^^*^P < 0.01; ^*^^*^^*^P < 0.001.

 It should be noted in Table 3 that for the 2 out of 18 tumors (ESCA and STAD) for which adding the connectivity score appears nominally statistically significant, these *P*-values remain above the Bonferroni corrected statistical significance threshold of 0.003 (i.e. 0.05 divided by 18). As the results in Table 3 were created using the drug signatures that combined the results of the 6- and 24-h drug perturbation gene expression experiments, as a sensitivity analysis we repeated the procedure using the connectivity scores calculated using the drug signatures based on only the drug perturbation gene expression experiments with 6-h duration (Supplementary Table S4) or 24-h duration (Supplementary Table S5). The median increases in *R*^2^ obtained by adding the connectivity score are only 0.01 and 0.02%, respectively. In addition, none of the *P*-values obtained go below the multiple testing corrected statistical significance threshold of 0.003.

## Discussion

The hypothesis that drugs can normalize gene expression changes induced by a disease such as cancer are therapeutically active in treating this disease is attractive and rational. However, our study shows that TSR as currently used in the discovery of novel drugs for the treatment of cancer mainly relies on selecting drugs that have general anti-proliferative effects rather than drugs that interact specifically with the transcriptome characteristic for the tumor type.

This is best illustrated using Figure 3. Genes that are downregulated in the presence of drugs that strongly reduce cell viability (i.e. with positive Spearman correlation as illustrated in Figure 2F) are on average upregulated in cancer. This holds true both for drug signatures generated using data from the LINCS experiments after 6 and 24 h of drug exposure. Gene set enrichment analysis showed that these genes are associated with the pathways ‘Cell cycle’, ‘DNA replication’, ‘Mismatch repair’, ‘Base excision repair’ and ‘Cellular senescence’, indicating that this inverse association is caused by a general anti-proliferation response, not the targeting of a specific pathway uniquely driving the tumor cells.

Indeed, after removing the effect of drug-induced decreased cell viability from the drug signatures by conditioning on effects seen in other cell lines at the same mnAUC, the connectivity scores are no longer predictive of drug effectivity (Figure 4). In addition, while the connectivity scores generated using the uncorrected drug signatures do have some predictive ability in predicting the average AUC of drugs in cell lines belonging to a specific tumor type (median *R*^2^ of 1.6%), this turns out to be negligible compared with simply using the average AUC of the drugs in other cell lines (median *R*^2^ of 95.5%). Furthermore, adding the connectivity score as a covariate to the average efficacy of the drugs in other cell lines only increases the median *R*^2^ by 0.01%, which is a statistically insignificant amount for 16 out of the 18 tumor types included in the connectivity score analyses. This is further evidence to support the hypothesis that the connectivity score using the uncorrected drug signatures only captures a fraction of the generic anti-proliferation potential of a drug and does not provide any increased specificity toward cancer sub(types), which would be useful to support a drug repurposing effort.

Previous systematic research reported much higher predictive ability using TSR and connectivity scores as compared with our estimates, namely, explained variabilities (*R*^2^) of 7% for invasive breast (*n* = 100 drugs), 26% for liver hepatocellular carcinoma (*n* = 24 drugs) and 16% for colon adenocarcinoma (*n* = 58 drugs) [6]. In our analysis, we found explained proportions of variance of <4% for these same three tumors. While our study uses different data (PRISM data set instead of ChEMBL), both studies in principle test the same hypothesis. To keep our methods consistent with the methods of Chen *et al*., we used the same method of calculating connectivity scores, similarly combined the 6- and 24-h drug signatures and in one of our four tested scenarios used the same log_2_-FC and a statistical significance cutoff (with identical results when testing the top 50, 100 or 150 most statistically significant genes of each tumor type). A difference is that in our study each drug’s effect on cell viability is based on the results of all cell lines belonging to a specific tumor type in the PRISM data set, whereas the study by Chen *et al*. selected a single cell line as representative for each tumor type. In addition, we tested 400+ drugs for each of the 18 tumor types, whereas Chen *et al*. only included 24–100 drugs. All aspects combined, we believe our study constitutes a more comprehensive effort to test the underlying TSR hypothesis by using more drug data, by varying the method of selecting the tumor signature genes and by combining the results of all cell lines belonging to particular tumor type.

A possible objection to our method of removing the effect of reduced cell viability from the drug signatures is that it might remove part of the more upstream (i.e. causal) effects contained in the drug signature as well. Many of the drugs decreasing cell viability will use different mechanisms of action and thus the expected correlation between mnAUC and gene expression of genes in upstream pathways will be relatively weak, compared with the correlation between mnAUC and genes in the downstream pathways associated with cell viability in the same direction. Therefore, the corrected drug signatures are still expected to contain upregulated and downregulated genes specific to their upstream, primary mechanism of action although the magnitude of the upregulation and downregulation might have been reduced. Despite this, it remains possible some upstream pathways may on average be affected similarly in response to drugs decreasing cell viability as when the pathway is directly targeted. While this possibility exists and may hold true for some pathways, previous research has demonstrated that many drug signatures belonging to the same drug class cluster together in statistical analyses and therefore do contain unique information [3]. Additionally, from Supplementary Figure S4 it can be seen that there is no statistically significant difference in the median number of DEG per drug signature between corrected and uncorrected data.

 The results from this paper are primarily relevant to the use of TSR for the purpose of finding new anticancer drugs. However, we suggest that our findings should serve as a more general caveat to the use of TSR outside of oncology that the relatively nonspecific method of attempting to reverse all DEG associated with a specific disease may not be optimal, and there should be an attempt to separate out the effect of different gene expression pathways and their upstream and downstream effects on the transcriptome.

Despite the presented evidence supporting a refined view of TSR efforts, we believe that TSR still has potential left to be useful as a drug repurposing method within oncology. For such efforts, we have three recommendations:

First, it seems necessary to use single-cell RNA-seq data instead of bulk RNA-seq data. Using bulk RNA-seq data is conceptually problematic because the gene expression of all cell types present in the sample is measured at the same time. Solid tumor samples consist of tumor cells intermixed with other cell types such as immune cells, endothelial cells and stromal cells, whereas bulk adjacent normal samples contain varying mixtures of normal cells, none of which may be of the cell type from which the first tumor cell originated. For example, clear cell renal cell carcinoma (ccRCC) tumors are believed to originate from proximal convoluted tubule of the nephron [16], implying that it would make the most sense to contrast the gene expression of ccRCC tumor cells to the gene expression of proximal tubules cells, preferably of the same patient.

Second, separating out the effect of driver events from passenger events is required. Tumor cells have to acquire many different driver events (e.g. mutations and copy number events) in specific sequences before they become numerous enough to cause symptoms and the patient is diagnosed with a tumor [17]. Because of increasing genetic instability along the way, tumor cells also acquire passenger events that do not increase tumor cell survivability, e.g. random mutations or genes that are co-amplified or co-deleted in copy number events. This makes the gene expression of each tumor clone unique, but only reversing the impact of driver events is expected to provide a therapeutic benefit. A statistical model containing all driver events and passenger events could separate out the gene expression impact of each, revealing which driver events have a gene expression signature potentially reversible by already available drugs. Other potentially useful approaches to select which genes are most therapeutically useful to target for the inversion of the gene expression are, e.g. network analysis [18], gene co-expression analysis [19, 20], causal analysis [21, 22] and graphical models [23]. As an alternative to or in addition to using clinical data, the effect of driver events can be experimentally determined using cell lines or patient-derived organoids modified with CRISPR [24], although this method has the downside of being less representative of the *in vivo*-situation lacking the interaction of the tumor cells with the tumor microenvironment.

Third, the scope of TSR could be broadened to non-tumor cell types present in the tumor microenvironment. Tumors survive by manipulating their environment to serve their needs, such as suppressing immune cells and stimulating endothelial cells to grow new blood vessels. Existing drugs that target the CTLA-4 receptor on T cells (e.g. ipilimumab) and VEGF receptor on cells of the vascular endothelium (e.g. sunitinib) already prove that this can be an effective strategy. For example, the ideal contrasts in these cases would be to compare the gene expression of inactivated immune cells in the tumor to those in the blood and cells in the vascular endothelium of the tumor to those in healthy tissues outside of the tumor. It would be especially interesting to do this for patients for whom therapy failed immediately or after the development of resistance, as the interactions between the tumor cells and non-tumor cells of interest are likely facilitated through as of yet understudied pathways which may be targetable by a drug already on the market.

We believe that future efforts taking these recommendations into account may help to improve the specificity of drug repurposing.

**Key Points**

- Transcriptome signature reversion (TSR) is a widely used computational method to find new uses for existing drugs, for example, against specific tumor types. It assumes that drug gene expression signatures that can reverse the gene expression signature of the malignant phenotype are more likely to be therapeutic in treating that phenotype.
- The connectivity score (measuring gene signature reversion), while statistically significant in most cases, only explains a median of 1.6% of the variation in the sensitivity of cell lines to drugs. Additionally, it does not provide any predictive benefit over the mean sensitivity measured using other cell lines not descended from the same tumor type.
- Removing the impact of decreased cell viability from the drug signatures completely nullified the predictive performance. This implies that the connectivity score as currently used only quantifies the generic anti-proliferative potential of drug, and cannot be used to reposition drugs against specific tumor types.
- We discuss several possible improvements on the current use of TSR, which may make it more useful for drug repositioning in the future.

## Supplementary Material

Supplemental_information_bbac490

## Data Availability

All code and data underlying this article are available in on GitLab, and can be accessed at https://gitlab.com/k.k.m.koudijs/TSR-comprehensive-validation.

## Author Biographies

**Karel K.M. Koudijs** is a PhD student of Leiden University Medical Center. He is working on computational drug repositioning.

**Stefan Böhringer** is an assistant professor of Leiden University Medical Center. He is a statistician who consults on ‘omics’ analyses.

**Henk-Jan Guchelaar** is a full professor of Leiden University Medical Center. He specializes in clinical pharmacy and toxicology with an interest in drug repositioning.

## References

[1] Koudijs KKM , Terwisscha van Scheltinga AGT, Böhringer S, et al. Transcriptome signature reversion as a method to reposition drugs against cancer for precision oncology. Cancer J 2019;25(2):116–20. 30896533 10.1097/PPO.0000000000000370

[2] Lamb J , Crawford ED, Peck D, et al. The Connectivity Map: using gene-expression signatures to connect small molecules, genes, and disease. Science 2006;313(5795):1929–35. 17008526 10.1126/science.1132939

[3] Subramanian A , Narayan R, Corsello SM, et al. A next generation Connectivity Map: L1000 platform and the first 1,000,000 profiles. Cell 2017;171(6):1437–52.e17. 29195078 10.1016/j.cell.2017.10.049 PMC5990023

[4] Bhat-Nakshatri P , Goswami CP, Badve S, et al. Identification of FDA-approved drugs targeting breast cancer stem cells along with biomarkers of sensitivity. Sci Rep 2013;3:2530. 23982413 10.1038/srep02530 PMC3965360

[5] Chen B , Wei W, Ma L, et al. Computational discovery of niclosamide ethanolamine, a repurposed drug candidate that reduces growth of hepatocellular carcinoma cells in vitro and in mice by inhibiting cell division cycle 37 signaling. Gastroenterology 2017;152(8):2022–36. 28284560 10.1053/j.gastro.2017.02.039 PMC5447464

[6] Chen B , Ma L, Paik H, et al. Reversal of cancer gene expression correlates with drug efficacy and reveals therapeutic targets. Nat Commun 2017;8:16022. 28699633 10.1038/ncomms16022 PMC5510182

[7] Kim IW , Jang H, Kim JH, et al. Computational drug repositioning for gastric cancer using reversal gene expression profiles. Sci Rep 2019;9(1):2660. 30804389 10.1038/s41598-019-39228-9 PMC6389943

[8] van Noort V , Schölch S, Iskar M, et al. Novel drug candidates for the treatment of metastatic colorectal cancer through global inverse gene-expression profiling. Cancer Res 2014;74(20):5690–9. 25038229 10.1158/0008-5472.CAN-13-3540

[9] Sirota M , Dudley JT, Kim J, et al. Discovery and preclinical validation of drug indications using compendia of public gene expression data. Sci Transl Med 2011;3(96):96ra77. 10.1126/scitranslmed.3001318 PMC3502016 21849665

[10] Chen HR , Sherr DH, Hu Z, et al. A network based approach to drug repositioning identifies plausible candidates for breast cancer and prostate cancer. BMC Med Genomics 2016;9(1):51. 27475327 10.1186/s12920-016-0212-7 PMC4967295

[11] Vásquez-Bochm LX , Velázquez-Paniagua M, Castro-Vázquez SS, et al. Transcriptome-based identification of lovastatin as a breast cancer stem cell-targeting drug. Pharmacol Rep 2019;71(3):535–44. 31026757 10.1016/j.pharep.2019.02.011

[12] Szalai B , Subramanian V, Holland CH, et al. Signatures of cell death and proliferation in perturbation transcriptomics data-from confounding factor to effective prediction. Nucleic Acids Res 2019;47(19):10010–26. 31552418 10.1093/nar/gkz805 PMC6821211

[13] Corsello SM , Nagari RT, Spangler RD, et al. Discovering the anti-cancer potential of non-oncology drugs by systematic viability profiling. Nat Cancer 2020;1(2):235–48. 32613204 10.1038/s43018-019-0018-6 PMC7328899

[14] Goldman MJ , Craft B, Hastie M, et al. Visualizing and interpreting cancer genomics data via the Xena platform. Nat Biotechnol 2020;38(6):675–8. 32444850 10.1038/s41587-020-0546-8 PMC7386072

[15] Chen EY , Tan CM, Kou Y, et al. Enrichr: interactive and collaborative HTML5 gene list enrichment analysis tool. BMC Bioinform 2013;14:128. 10.1186/1471-2105-14-128 PMC3637064 23586463

[16] Frew IJ , Moch H. A clearer view of the molecular complexity of clear cell renal cell carcinoma. Annu Rev Pathol 2015;10:263–89. 25387056 10.1146/annurev-pathol-012414-040306

[17] Turajlic S , Xu H, Litchfield K, et al. Deterministic evolutionary trajectories influence primary tumor growth: TRACERx renal. Cell 2018;173(3):595–610.e11. 29656894 10.1016/j.cell.2018.03.043 PMC5938372

[18] Chen HM , MacDonald JA. Network analysis of TCGA and GTEx gene expression datasets for identification of trait-associated biomarkers in human cancer. STAR Protoc 2022;3(1):101168. 35199033 10.1016/j.xpro.2022.101168 PMC8841814

[19] van Dam S , Võsa U, van der Graaf A, et al. Gene co-expression analysis for functional classification and gene-disease predictions. Brief Bioinform 2018;19(4):575–92. 28077403 10.1093/bib/bbw139 PMC6054162

[20] Niu X , Zhang J, Zhang L, et al. Weighted gene co-expression network analysis identifies critical genes in the development of heart failure after acute myocardial infarction. Front Genet 2019;10:1214. 31850068 10.3389/fgene.2019.01214 PMC6889910

[21] Lee E , Cho S, Kim K, et al. An integrated approach to infer causal associations among gene expression, genotype variation, and disease. Genomics 2009;94(4):269–77. 19540336 10.1016/j.ygeno.2009.06.002

[22] Park YP , Kellis M. CoCoA-diff: counterfactual inference for single-cell gene expression analysis. Genome Biol 2021;22(1):228. 34404460 10.1186/s13059-021-02438-4 PMC8369635

[23] Hartemink AJ , Gifford DK, Jaakkola TS, et al. Using graphical models and genomic expression data to statistically validate models of genetic regulatory networks. Pac Symp Biocomput 2001;422–33. 11262961 10.1142/9789814447362_0042

[24] Okamoto T , Natsume Y, Yamanaka H, et al. A protocol for efficient CRISPR-Cas9-mediated knock-in in colorectal cancer patient-derived organoids. STAR Protoc 2021;2(4):100780. 34585151 10.1016/j.xpro.2021.100780 PMC8455475

