# Supplementary material for: Validation of transcriptome signature reversion for drug repurposing in oncology
Source: Brief Bioinform. 2022 Nov 29;24(1):bbac490. doi: 10.1093/bib/bbac490 (PMC9851289; doi:10.1093/bib/bbac490)
Supplement: Supplemental_information_bbac490 [file supplemental_information_bbac490.docx]

**SUPPLEMENTAL INFORMATION to**

Validation of transcriptome signature reversion (TSR) for drug repurposing in oncology

Karel K. M. Koudijs^1^, Stefan Böhringer^1,2^, Henk-Jan Guchelaar^1*^**Affiliations:**

^1^ Department of Clinical Pharmacy and Toxicology, Leiden University Medical Center; 2333 ZA Leiden, The Netherlands.

^2^ Department of Biomedical Data Sciences, Leiden University Medical Center; 2333 ZA Leiden, The Netherlands.

* Corresponding author e-mail address: [h.j.guchelaar@lumc.nl](mailto:h.j.guchelaar@lumc.nl)

**Table of contents:**

| Item | Description | Page |
| --- | --- | --- |
| Table S1 | Number of tumor and adjacent normal samples of each included TCGA project, including how many cell lines in PRISM each tumor type could be mapped to and the number of differentially expressed genes which exceed the cutoff from the 2017 publication of Bin Chen et al. | 2 |
| Table S2 | Spearman correlation between DEG tumor versus normal and Spearman Rho between the mnAUC with the DEG after 6h, 24h and 6h + 24h. | 3 |
| Table S3 | Top 25 genes most strongly downregulated when exposed to drugs which reduce cell viability. | 4 |
| Table S4 | Percentage of variation explained in the mean AUC of cell lines belonging to a specific tumor type by 2 different linear models using the connectivity scores calculated with the uncorrected 6h drug signatures. | 5 |
| Table S5 | Percentage of variation explained in the mean AUC of cell lines belonging to a specific tumor type by 2 different linear models using the connectivity scores calculated with the uncorrected 24h drug signatures. | 5 |
| Figure S1 | Visualization of cutoff for low expressed genes from TCGA tumor types. | 6 |
| Figure S2 | Graphical illustration of the correction method used to filter out the effect of drug-induced decreased cell viability from the specific effect of the drug itself. | 7 |
| Figure S3 | Example of the original connectivity score and the new connectivity score calculated on the same data using 50 genes as input. | 8 |
| Figure S4 | Percentage of genes differentially expressed after exposure to drug plotted against the mnAUC of the drug. | 9 |

**Supplementary Table S1.** Number of primary solid tumor (sample type code 01) and solid tissue normal (sample type code 11) RNA-seq samples from TCGA, including how many cell lines in PRISM each tumor type could be mapped to and the number of differentially expressed genes which exceed the cutoff from the 2017 publication of Bin Chen et al.

| TCGA project | Description | Number of tumor samples | Number of normal samples | Mapping to PRISM cell line origin of tissue (N = Resulting number of different cell lines) | Number of genes > 1.5 log_2_FC & adj. P < 0.001 shared with LINCS |
| --- | --- | --- | --- | --- | --- |
| ACC | Adrenocortical carcinoma | 79 | 0 | - | - |
| BLCA | Bladder Urothelial Carcinoma | 411 | 19 | urinary_tract > all (N = 23) | 91 |
| BRCA | Breast invasive carcinoma | 1,097 | 113 | breast > all (N = 22) | 85 |
| CESC | Cervical squamous cell carcinoma and endocervical adenocarcinoma | 304 | 3 | - | 109 |
| CHOL | Cholangiocarcinoma | 36 | 9 | bile_duct > cholangiocarcinoma (N = 6) | 162 |
| COAD | Colon adenocarcinoma | 469 | 41 | colorectal > all (N = 27) | 67 |
| DLBC | Lymphoid Neoplasm Diffuse Large B-cell Lymphoma | 48 | 0 |  | - |
| ESCA | Esophageal carcinoma | 161 | 11 | esophagus > all (N = 24) | 40 |
| GBM | Glioblastoma multiforme | 155 | 5 | central_nervous_system > glioma (N = 31) | 176 |
| HNSC | Head and Neck squamous cell carcinoma | 500 | 44 | - | 48 |
| KICH | Kidney Chromophobe | 65 | 24 | kidney > all (N = 16) | 116 |
| KIRC | Kidney renal clear cell carcinoma | 534 | 72 | kidney > all (N = 16) | 90 |
| KIRP | Kidney renal papillary cell carcinoma | 288 | 32 | kidney > all (N = 16) | 66 |
| LGG | Brain Lower Grade Glioma | 511 | 0 | - | - |
| LIHC | Liver hepatocellular carcinoma | 371 | 50 | liver > all (N = 17) | 76 |
| LUAD | Lung adenocarcinoma | 524 | 59 | lung > lung_NSC > lung_adenocarcinoma (N = 46) | 89 |
| LUSC | Lung squamous cell carcinoma | 501 | 49 | lung > lung_NSC > lung_squamous (N = 17) | 150 |
| MESO | Mesothelioma | 86 | 0 | - | - |
| OV | Ovarian serous cystadenocarcinoma | 374 | 0 | - | - |
| PAAD | Pancreatic adenocarcinoma | 177 | 4 | pancreas > all (N = 33) | 0 |
| PCPG | Pheochromocytoma and Paraganglioma | 178 | 3 | - | 93 |
| PRAD | Prostate adenocarcinoma | 498 | 52 | prostate > all (N = 4) | 24 |
| READ | Rectum adenocarcinoma | 166 | 10 | - | 63 |
| SARC | Sarcoma | 259 | 2 | soft_tissue > all (N = 4) | 10 |
| SKCM | Skin Cutaneous Melanoma | 103 | 0 | - | - |
| STAD | Stomach adenocarcinoma | 375 | 32 | gastric > gastric_adenocarcinoma (N = 14) | 63 |
| TGCT | Testicular Germ Cell Tumors | 150 | 0 | - |  |
| THCA | Thyroid carcinoma | 502 | 58 | thyroid > all (N = 11) | 37 |
| THYM | Thymoma | 119 | 2 | - | 3 |
| UCEC | Uterine Corpus Endometrial Carcinoma | 547 | 35 | uterus_endometrium > all (N = 21) | 118 |
| UCS | Uterine Carcinosarcoma | 56 | 0 | - | - |
| UVM | Uveal Melanoma | 80 | 0 | - | - |

**Supplementary Table S2.** Spearman correlation between DEG tumor versus normal and Spearman Rho between the mnAUC with the DEG after 6h, 24h and 6h + 24h. Legend: * = P < 0.05, ** = P < 0.01 and *** = P < 0.001.

| TCGA project | Rho calculated using DEG after 6h drug exposure | | Rho calculated using DEG after 24h drug exposure | | Rho calculated using DEG after 6h + 24h drug exposure | |
| --- | --- | --- | --- | --- | --- | --- |
| BLCA | +0.24 (P = 1 x 10^-13^) | *** | +0.26 (P = 4 x 10^-16^) | *** | +0.28 (P = 4 x 10^-18^) | *** |
| BRCA | +0.17 (P = 2 x 10^-07^) | *** | +0.21 (P = 1 x 10^-10^) | *** | +0.21 (P = 2 x 10^-11^) | *** |
| CESC | +0.16 (P = 6 x 10^-07^) | *** | +0.19 (P = 6 x 10^-09^) | *** | +0.20 (P = 4 x 10^-10^) | *** |
| CHOL | +0.08 (P = 0.01) | * | +0.04 (P = 0.18) |  | +0.06 (P = 0.07) |  |
| COAD | +0.17 (P = 2 x 10^-07^) | *** | +0.18 (P = 4 x 10^-08^) | *** | +0.18 (P = 1 x 10^-08^) | *** |
| ESCA | +0.13 (P = 9 x 10^-05^) | *** | +0.16 (P = 9 x 10^-07^) | *** | +0.16 (P = 6 x 10^-07^) | *** |
| GBM | +0.13 (P = 0.0001) | *** | +0.18 (P = 3 x 10^-08^) | *** | +0.18 (P = 1 x 10^-08^) | *** |
| HNSC | +0.07 (P = 0.03) | * | +0.10 (P = 0.003) | ** | +0.09 (P = 0.004) | ** |
| KICH | +0.11 (P = 0.0009) | *** | +0.07 (P = 0.03) | * | +0.08 (P = 0.01) | * |
| KIRC | +0.02 (P = 0.45) |  | +0.04 (P = 0.27) |  | +0.04 (P = 0.24) |  |
| KIRP | +0.13 (P = 0.0001) | *** | +0.14 (P = 9 x 10^-06^) | *** | +0.15 (P = 5 x 10^-06^) | *** |
| LIHC | +0.24 (P = 2 x 10^-14^) | *** | +0.22 (P = 7 x 10^-12^) | *** | +0.24 (P = 2 x 10^-14^) | *** |
| LUAD | +0.21 (P = 4 x 10^-11^) | *** | +0.25 (P = 3 x 10^-15^) | *** | +0.26 (P = 2 x 10^-16^) | *** |
| LUSC | +0.25 (P = 8 x 10^-15^) | *** | +0.28 (P = 3 x 10^-18^) | *** | +0.29 (P = 5 x 10^-20^) | *** |
| PAAD | +0.12 (P = 0.0001) | *** | +0.13 (P = 4 x 10^-05^) | *** | +0.14 (P = 1 x 10^-05^) | *** |
| PCPG | +0.05 (P = 0.15) |  | +0.05 (P = 0.12) |  | +0.05 (P = 0.13) |  |
| PRAD | +0.21 (P = 7 x 10^-11^) | *** | +0.22 (P = 2 x 10^-11^) | *** | +0.23 (P = 7 x 10^-13^) | *** |
| READ | +0.17 (P = 3 x 10^-07^) | *** | +0.15 (P = 5 x 10^-06^) | *** | +0.16 (P = 5 x 10^-07^) | *** |
| SARC | +0.14 (P = 7 x 10^-06^) | *** | +0.16 (P = 1 x 10^-06^) | *** | +0.16 (P = 4 x 10^-07^) | *** |
| STAD | +0.2 (P = 9 x 10^-10^) | *** | +0.19 (P = 2 x 10^-09^) | *** | +0.21 (P = 6 x 10^-11^) | *** |
| THCA | +0.02 (P = 0.56) |  | +0.06 (P = 0.05) |  | +0.05 (P = 0.10) |  |
| THYM | +0.08 (P = 0.01) | * | +0.09 (P = 0.004) | ** | +0.1 (P = 0.003) | ** |
| UCEC | +0.15 (P = 3 x 10^-06^) | *** | +0.2 (P = 6 x 10^-10^) | *** | +0.21 (P = 2 x 10^-10^) | *** |

**Supplementary Table S3.** Top 25 genes most strongly downregulated when exposed to drugs which reduce cell viability (as evidenced by the strong Spearman correlation coefficients (Rho). All Rho P-values are below 10^-16^.

| Gene | Rho | Gene description |
| --- | --- | --- |
| MCM3 | +0.56 | minichromosome maintenance complex component 3 |
| POLE2 | +0.54 | DNA polymerase epsilon 2, accessory subunit |
| TSEN2 | +0.53 | tRNA splicing endonuclease subunit 2 |
| MSH6 | +0.53 | mutS homolog 6 |
| CDK1 | +0.52 | cyclin dependent kinase 1 |
| MRPL12 | +0.50 | mitochondrial ribosomal protein L12 |
| LSM6 | +0.49 | LSM6 homolog, U6 small nuclear RNA and mRNA degradation associated |
| MELK | +0.48 | maternal embryonic leucine zipper kinase |
| CHEK2 | +0.47 | checkpoint kinase 2 |
| CCNA2 | +0.47 | cyclin A2 |
| TOP2A | +0.47 | DNA topoisomerase II alpha |
| CCNF | +0.47 | cyclin F |
| HDAC2 | +0.46 | histone deacetylase 2 |
| HMGA2 | +0.46 | high mobility group AT-hook 2 |
| PCNA | +0.45 | proliferating cell nuclear antigen |
| CDCA4 | +0.45 | cell division cycle associated 4 |
| PIP4K2B | +0.45 | phosphatidylinositol-5-phosphate 4-kinase type 2 beta |
| DCK | +0.45 | deoxycytidine kinase |
| EXOSC4 | +0.45 | exosome component 4 |
| UBE2C | +0.45 | ubiquitin conjugating enzyme E2 C |
| TMEM109 | +0.44 | transmembrane protein 109 |
| SLC37A4 | +0.44 | solute carrier family 37 member 4 |
| MAT2A | +0.44 | methionine adenosyltransferase 2A |
| BRCA1 | +0.44 | BRCA1 DNA repair associated |
| RUVBL1 | +0.43 | RuvB like AAA ATPase 1 |

**Supplementary Table S4.** Percentage of variation explained in the mean AUC of cell lines belonging to a specific tumor type by 2 different linear models using the connectivity scores calculated with the uncorrected 6h drug signatures. If the correlation coefficient of the connectivity score was negative (i.e. opposite the expected direction) the P-values were replaced with 1. *Legend*: * = P < 0.05, ** = P < 0.01 and *** = P < 0.001.

| TCGA project | Model 1: Connectivity score as covariate  (R^2^ and P-value versus null model) | | Model 2: Mean AUC calculated using other cell lines as covariate (R^2^ and P-value versus  null model) | | Model 3: Connectivity score added to model 2  (Increase in R^2^ and P-value versus model 2) | |
| --- | --- | --- | --- | --- | --- | --- |
| BLCA | 4.2% (P = 3 x 10^-6^) | *** | 97.6% (P < 1 x 10^-240^) | *** | 0.0008% (P = 1) |  |
| BRCA | 2.44% (P = 0.0004) | *** | 96.3% (P < 1 x 10^-240^) | *** | 0.02% (P = 0.09) |  |
| CHOL | 0.11% (P = 1) |  | 91.7% (P < 1 x 10^-240^) | *** | 0.05% (P = 0.08) |  |
| COAD | 4.41% (P = 2 x 10^-6^) | *** | 95.5% (P < 1 x 10^-240^) | *** | 0.006% (P = 0.42) |  |
| ESCA | 0.57% (P = 0.09) |  | 96.8% (P < 1 x 10^-240^) | *** | 0.03% (P = 0.03) | * |
| GBM | 3.55% (P = 2 x 10^-5^) | *** | 96.9% (P < 1 x 10^-240^) | *** | 0.0007% (P = 1) |  |
| KICH | 0.02% (P = 1) |  | 88.8% (P < 1 x 10^-240^) | *** | 0.02% (P = 0.34) |  |
| KIRC | 0.41% (P = 1) |  | 88.8% (P < 1 x 10^-240^) | *** | 0.02% (P = 1) |  |
| KIRP | 0.94% (P = 0.03) | * | 88.8% (P < 1 x 10^-240^) | *** | 0.06% (P = 0.11) |  |
| LIHC | 4.95% (P = 4 x 10^-7^) | *** | 95.2% (P < 1 x 10^-240^) | *** | 0.02% (P = 0.20) |  |
| LUAD | 2.77% (P = 0.0002) | *** | 98.7% (P < 1 x 10^-240^) | *** | 0.002% (P = 1) |  |
| LUSC | 3.29% (P = 4 x 10^-5^) | *** | 97.1% (P < 1 x 10^-240^) | *** | 0.007% (P = 1) |  |
| PAAD | 1.45% (P = 0.007) | ** | 97.3% (P < 1 x 10^-240^) | *** | 0.003% (P = 0.45) |  |
| PRAD | 3.81% (P = 1 x 10^-5^) | *** | 89.5% (P < 1 x 10^-240^) | *** | 0.01% (P = 0.50) |  |
| SARC | 0.15% (P = 0.39) |  | 90.1% (P < 1 x 10^-240^) | *** | 0.05% (P = 1) |  |
| STAD | 3.60% (P = 2 x 10^-5^) | *** | 95.3% (P < 1 x 10^-240^) | *** | 0.05% (P = 0.03) | * |
| THCA | 0.08% (P = 1) |  | 95.5% (P < 1 x 10^-240^) | *** | 0.01% (P = 1) |  |
| UCEC | 3.03% (P = 8 x 10^-5^) | *** | 96.4% (P < 1 x 10^-240^) | *** | 0.0008% (P = 1) |  |
| Median: | 2.6% |  | 95.5% |  | 0.013% |  |

**Supplementary Table S5.** Percentage of variation explained in the mean AUC of cell lines belonging to a specific tumor type by 2 different linear models using the connectivity scores calculated with the uncorrected 24h drug signatures. If the correlation coefficient of the connectivity score was negative (i.e. opposite the expected direction) the P-values were replaced with 1. *Legend*: * = P < 0.05, ** = P < 0.01 and *** = P < 0.001.

| TCGA project | Model 1: Connectivity score as covariate  (R^2^ and P-value versus null model) | | Model 2: Mean AUC calculated using other cell lines as covariate (R^2^ and P-value versus  null model) | | Model 3: Connectivity score added to model 2  (Increase in R^2^ and P-value versus model 2) | |
| --- | --- | --- | --- | --- | --- | --- |
| BLCA | 7.38% (P = 5 x 10^-10^) | *** | 97.6% (P < 1 x 10^-240^) | *** | 0.01% (P = 0.12) |  |
| BRCA | 4.01% (P = 5 x 10^-6^) | *** | 96.3% (P < 1 x 10^-240^) | *** | 0.002% (P = 1) |  |
| CHOL | 1.04% (P = 0.02) | * | 91.7% (P < 1 x 10^-240^) | *** | 0.1% (P = 0.01) | * |
| COAD | 4.56% (P = 1 x 10^-6^) | *** | 95.5% (P < 1 x 10^-240^) | *** | 0.02% (P = 0.1) |  |
| ESCA | 2.79% (P = 0.0002) | *** | 96.8% (P < 1 x 10^-240^) | *** | 0.03% (P = 0.02) | * |
| GBM | 7.78% (P = 1 x 10^-10^) | *** | 96.9% (P < 1 x 10^-240^) | *** | 0.002% (P = 1) |  |
| KICH | 0.008% (P = 0.843) |  | 88.8% (P < 1 x 10^-240^) | *** | 0.07% (P = 0.08) |  |
| KIRC | 0.11% (P = 0.456) |  | 88.8% (P < 1 x 10^-240^) | *** | 0.001% (P = 1) |  |
| KIRP | 3.18% (P = 5 x 10^-5^) | *** | 88.8% (P < 1 x 10^-240^) | *** | 0.03% (P = 1) |  |
| LIHC | 2.43% (P = 0.0004) | *** | 95.2% (P < 1 x 10^-240^) | *** | 0.00001% (P = 0.99) |  |
| LUAD | 6.01% (P = 2 x 10^-8^) | *** | 98.7% (P < 1 x 10^-240^) | *** | 0.002% (P = 1) |  |
| LUSC | 4.79% (P = 6 x 10^-7^) | *** | 97.1% (P < 1 x 10^-240^) | *** | 0.003% (P = 0.49) |  |
| PAAD | 3.99% (P = 6 x 10^-6^) | *** | 97.3% (P < 1 x 10^-240^) | *** | 0.003% (P = 1) |  |
| PRAD | 2.57% (P = 0.0002) | *** | 89.5% (P < 1 x 10^-240^) | *** | 0.05% (P = 1) |  |
| SARC | 1.83% (P = 0.002) | ** | 90.1% (P < 1 x 10^-240^) | *** | 0.05% (P = 1) |  |
| STAD | 5.73% (P = 5 x 10^-8^) | *** | 95.3% (P < 1 x 10^-240^) | *** | 0.05% (P = 0.02) | * |
| THCA | 0.55% (P = 0.09) |  | 95.5% (P < 1 x 10^-240^) | *** | 0.006% (P = 1) |  |
| UCEC | 2.59% (P = 0.0003) | *** | 96.4% (P < 1 x 10^-240^) | *** | 0.05% (P = 1) |  |
| Median: | 2.99% |  | 95.5% |  | 0.02% |  |


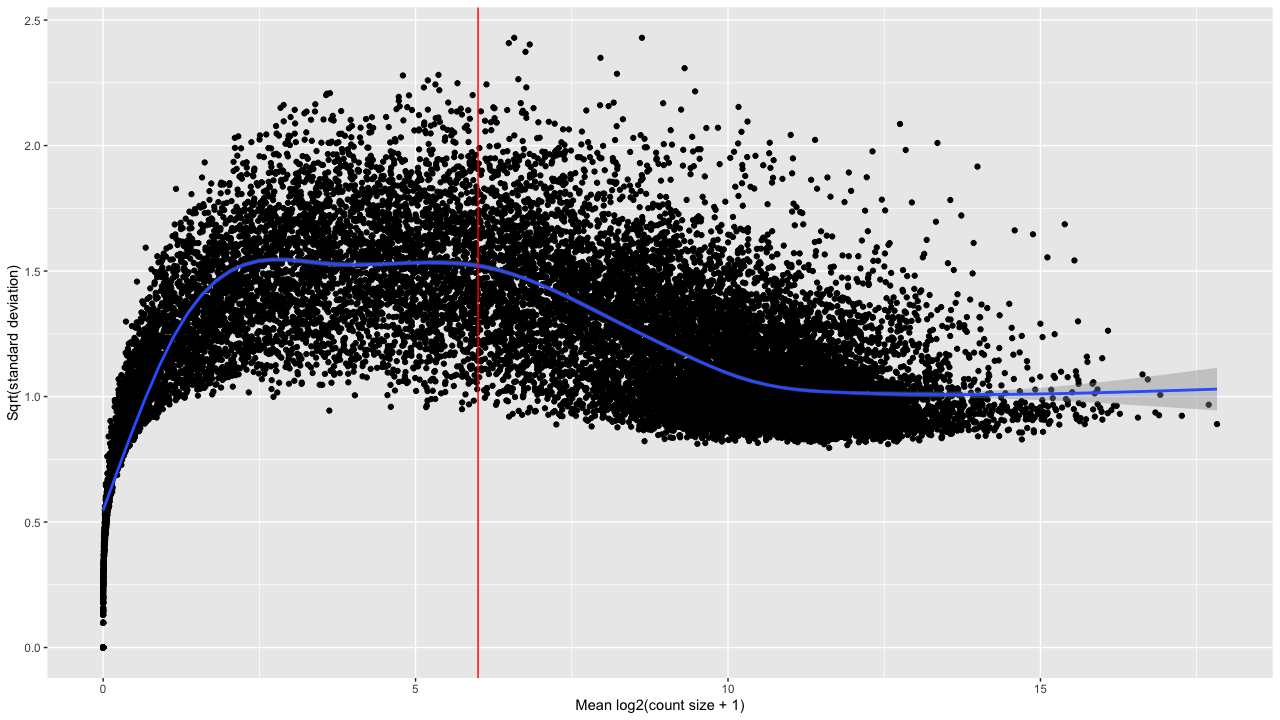


**Supplementary Figure S1.** Visualization of cutoff for low expressed genes from TCGA tumor types. The red line symbolizes the cutoff of a mean log_2_(counts + 1) > 6, above which the variation of the genes starts to decrease. After filtering, 13,386 genes remained.


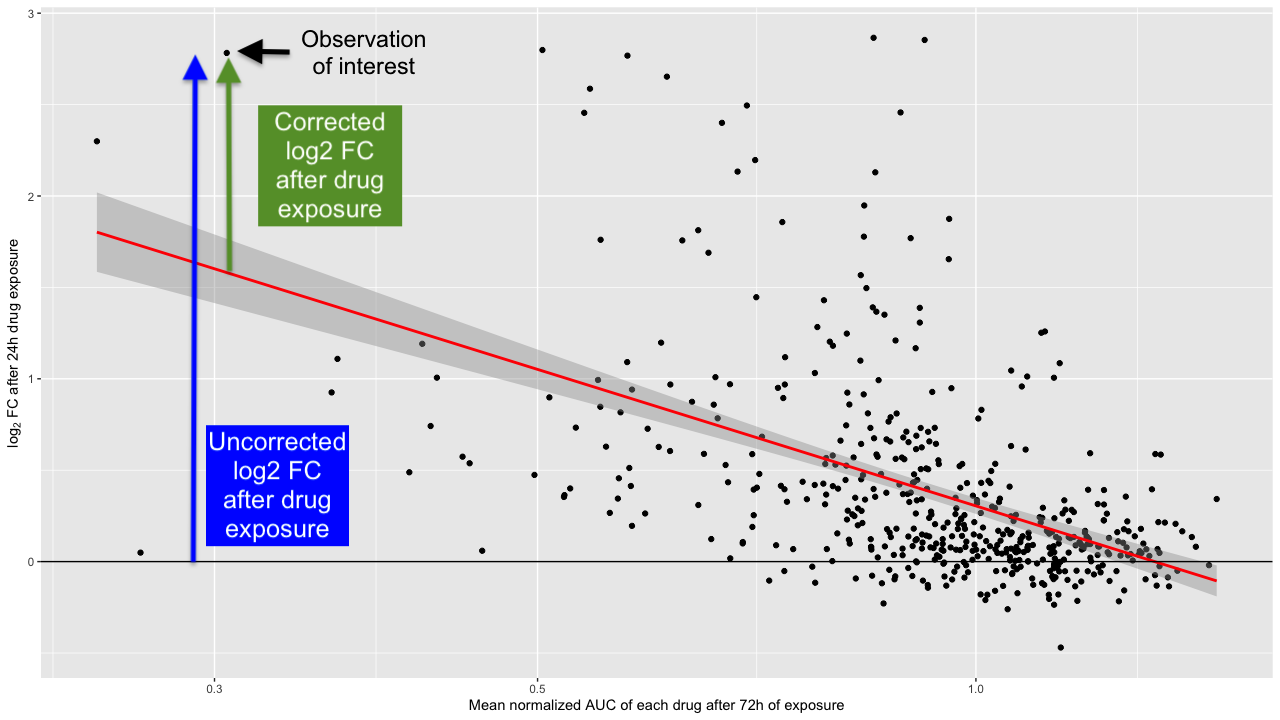


**Supplementary Figure S2.** Graphical illustration of the correction method used to filter out the effect of drug-induced decreased cell viability from the specific effect of the drug itself. The red line is the linear model fit, the X-axis has logarithmic scale.


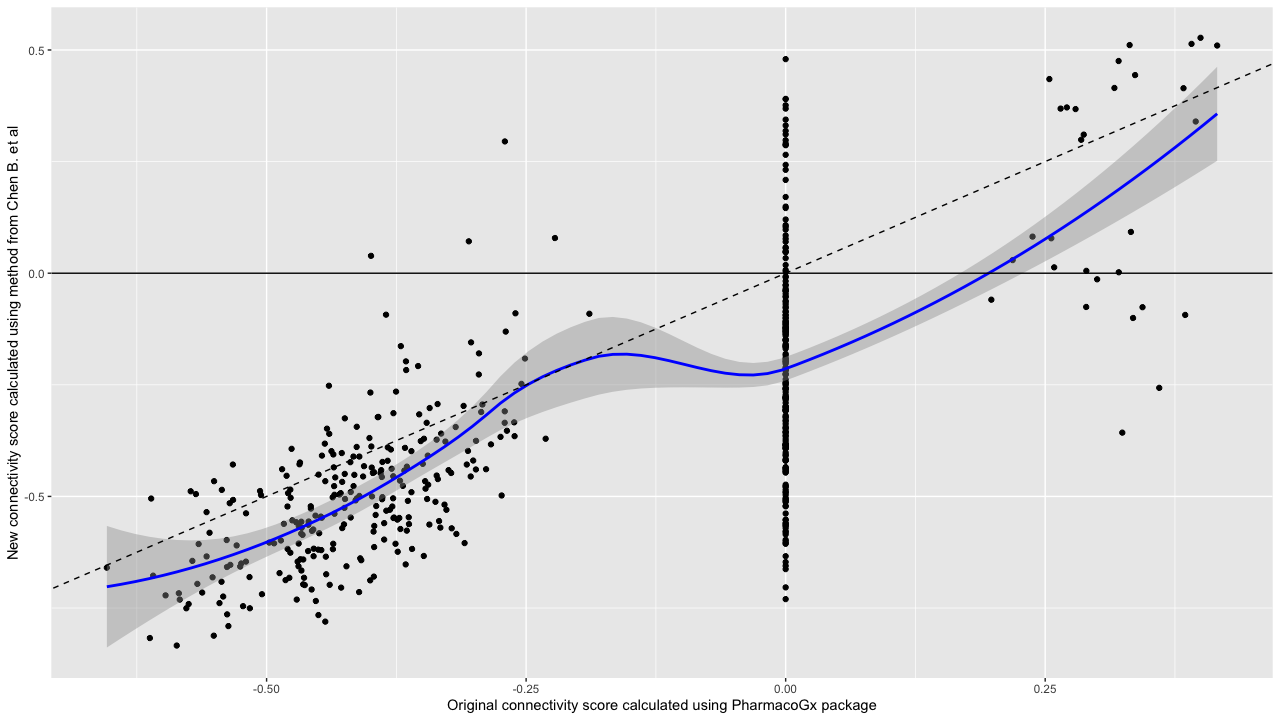

 **Supplementary Figure S3.** Example of the original connectivity score and the new connectivity score calculated on the same data using 50 genes as input. The dashed black line is the identity line, i.e. Y = X and the solid blue line represents the LOESS smoother. On average the connectivity score methods agree but there’s substantial variance in the exact value of the connectivity score. Furthermore, the original method produces a lot of connectivity scores of 0 as was reported in the paper of Chen B. et al.


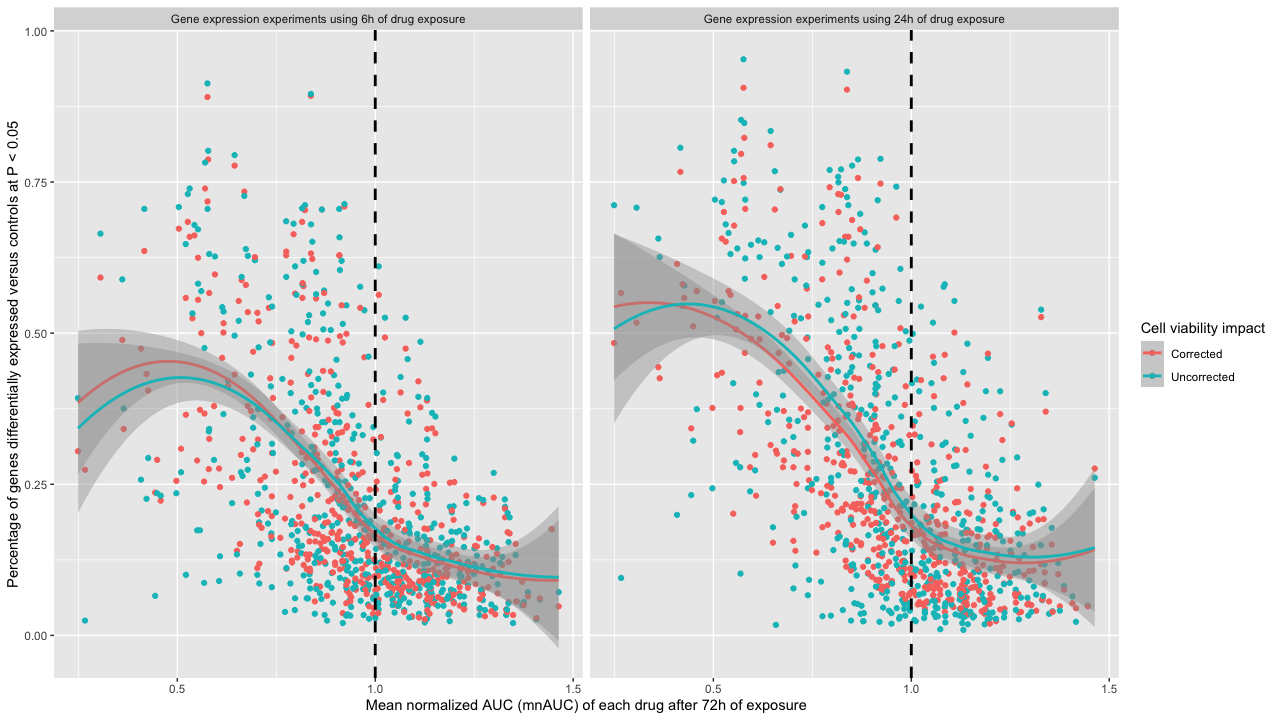


**Supplementary Figure S4.** Percentage of genes differentially expressed after exposure to drug plotted against the mnAUC of the drug. The red and turquoise lines are LOESS smoothers, the vertical black dashed line separates drugs which decrease cell viability (mnAUC < 1) from those who do not (mnAUC > 1). There is almost no difference in the number of statistically significant genes between corrected and uncorrected drug signatures. The biggest contrast is between drug signatures with a mnAUC > 1 and a mnAUC < 1: a mean of 30% versus a mean of 13% after 6h of drug exposure and a mean of 33% versus a mean of 14% after 24h of drug exposure.
